# Central and Peripheral Neuromuscular Adaptations to Ageing

**DOI:** 10.3390/jcm9030741

**Published:** 2020-03-09

**Authors:** Riccardo Borzuola, Arrigo Giombini, Guglielmo Torre, Stefano Campi, Erika Albo, Marco Bravi, Paolo Borrione, Chiara Fossati, Andrea Macaluso

**Affiliations:** 1 Department of Movement, Human and Health Sciences, University of Rome “Foro Italico”, 00135 Rome, Italy; riccardo.borzuola@uniroma4.it (R.B.); arrigo.giombini@uniroma4.it (A.G.); paolo.borrione@uniroma4.it (P.B.); chiara.fossati@uniroma4.it (C.F.); andrea.macaluso@uniroma4.it (A.M.); 2 Department of Orthopaedic And Trauma Surgery, Campus Bio-Medico University of Rome, 00128 Rome, Italy; s.campi@unicampus.it (S.C.); e.albo@unicampus.it (E.A.); 3 Department of Physical Medicine and Rehabilitation, Campus Bio-Medico University of Rome, 00128 Rome, Italy; m.bravi@unicampus.it

**Keywords:** aging, cerebral cortex, dynapenia, elderly, motor unit, muscle strength, neural, neuroplasticity, sarcopenia

## Abstract

Ageing is accompanied by a severe muscle function decline presumably caused by structural and functional adaptations at the central and peripheral level. Although researchers have reported an extensive analysis of the alterations involving muscle intrinsic properties, only a limited number of studies have recognised the importance of the central nervous system, and its reorganisation, on neuromuscular decline. Neural changes, such as degeneration of the human cortex and function of spinal circuitry, as well as the remodelling of the neuromuscular junction and motor units, appear to play a fundamental role in muscle quality decay and culminate with considerable impairments in voluntary activation and motor performance. Modern diagnostic techniques have provided indisputable evidence of a structural and morphological rearrangement of the central nervous system during ageing. Nevertheless, there is no clear insight on how such structural reorganisation contributes to the age-related functional decline and whether it is a result of a neural malfunction or serves as a compensatory mechanism to preserve motor control and performance in the elderly population. Combining leading-edge techniques such as high-density surface electromyography (EMG) and improved diagnostic procedures such as functional magnetic resonance imaging (fMRI) or high-resolution electroencephalography (EEG) could be essential to address the unresolved controversies and achieve an extensive understanding of the relationship between neural adaptations and muscle decline.

## 1. Introduction

Ageing is associated with loss in muscle mass and strength. Originally, scientists conceptualised the term “sarcopenia” to describe the age-related loss of skeletal muscle mass [1,2]. Others coined the term “dynapenia” to indicate the decline in muscle strength [3,4,5,6]. More recently, numerous investigators introduced the term “muscle quality” to describe the relationship between muscle strength and size in older adults [7,8,9,10]. However, whereas the large majority of the research has focused on the intrinsic skeletal muscle properties and mechanisms to explain muscle weakness in older adults, relatively little recognition has been given to the potential role of the central nervous system as a fundamental component of the decline in muscle function [11].

Researchers have suggested that the age-related decay in muscle quality can be attributed to many neural factors, including a decline in the function of the human cortex, spinal cord and neuromuscular junction [12]. Investigating the role of neural factors in preserving muscle properties and contraction capacities is key to developing a more comprehensive understanding of the causes leading to the decline in muscle function occurring at advancing age. In this review, we highlight the neurological and neuromuscular adaptations that primarily cause impairments in the skeletal muscle function and performance of the aged population.

## 2. Supraspinal Age-Related Adaptations

Premotor and primary motor cortical areas comprise a large number of excitatory and inhibitory neurons, including glutamatergic neurons, axonal projections and pyramidal neurons [13]. Besides projecting to the cortical areas of the central nervous system, these neurons form a long axonal connection with the lower motor neurons in the ventral horn of the spinal cord [13]. Several studies have shown that the premotor and the primary motor cortex (M1) incur cortical atrophy [14,15,16,17]. The total volume of the cortical area has been shown to decrease (between 4% and 16%) with age [14,15,16]. In the past, it was commonly assumed that this was attributed to a decrease in the number of M1 neurons during ageing. However, recent evidence suggested that, rather than a numerical decay in cortical neurons, M1 cortex decline is related to a volumetric reduction in premotor and primary motor neuron cell body size and synaptic density [16,17,18]. This adaptation has been referred to as neural atrophy [19]. A study on cadaveric dissections [20] demonstrated that the elderly population had an average of 43% volumetric reduction in the motor cortex cell body size compared to young adults. These findings have been more recently supported by studies performed on living humans using high-resolution magnetic resonance. Salat et al. [17] found that the cerebral cortex, including M1 cortex, incurs a substantial reduction in volume due to the morphometric changes in the neural cells. Several studies evidenced a strong correlation between cortical atrophy and fine movements, such as mirror drawing [21]. Other motor tasks appear to be substantially influenced by grey matter atrophy. Rosano et al. [22] reported a decline in gait performance during GaitMat walking proportional to the decrease in grey matter volume. Similarly, Sridharan et al. [23] illustrated a strong correlation between lower grey matter volumes and impaired reaching movements in aged rhesus monkeys.

Neural atrophy, however, is not the only morphometric adaptation that occurs during ageing. A major alteration involves the cortical white matter, which exhibits a significant decrease in subjects over 65 years old [24,25]. White matter is predominantly made up of glial cells and myelinated axons, and it is responsible for the cortico-cortical and cortico-spinal connectivity. Cross-sectional studies indicate a profound disruption in the integrity of the white matter [25], which declines with age at an average rate of 2.5% every ten years [26,27]. The experimental investigation of Marner et al. [28] indicated that myelinated nerve fibre length in the white matter significantly decreased in aged population compared to younger individuals. Age-related morphometric adaptations of the motor cortex may therefore carry a considerable effect on the connectivity within the cortical areas as well as between the cortex and the rest of the central nervous system [29]. Several studies have indicated that a decrease in white matter integrity is associated with slower motor performances on interhemispheric transfer task such as alternating finger tapping [30] and other fine finger movement tasks [31]. Zahr et al. [32] demonstrated that greater white matter integrity correlates with improved motor performance for both fine and gross motor skills. Other researchers indicated that white matter hyperintensities lead to poorer stability, expressed as greater sway path and impaired static balance [33].

Age-related decline in motor functions has also been imputed to impaired neurotransmission, which is regulated by neurochemical factors. Evidence suggests that alterations in neurotransmitters (NT) and their specific receptors are directly associated with impairments of the cognitive and motor function in the elderly population. Abnormalities in the NT-regulated systems such as serotonergic [34,35], cholinergic [36], adrenergic [35], dopaminergic, GABAergic and glutamatergic [37,38,39,40] have been highlighted in ageing individuals. Although a comprehensive understanding of how each system responds to ageing remains unclear, several investigators suggested that the interaction between glutamate, dopamine and GABA plays a critical role in the decline of cortical and motor functions in the elderly [40]. The NT-regulated systems’ alterations have been associated with increased neural noise, which has been defined as a random background activity in the brain signal [11].

Failures of the glutamatergic system have been ascribed as one of the neurochemical mechanisms responsible for the increase in neural noise since glutamate is directly involved in the modulation of the excitatory inputs in the central nervous system. The study of Arnth-Jensen et al. [41] demonstrated that ageing is accompanied by a significant reduction in the glutamate uptake. They found an excessive quantity of extracellular glutamate around cortical neurons and observed a significant increase in neural noise which is expressed by abnormal and unpredictable neural background activity even during simple motor tasks.

Furthermore, evidence exists that increased neural noise can be attributed to a decline of the dopaminergic system [42]. A previous investigation evidenced a considerable loss of dopamine transporters in the central nervous system with a 6.6% decay per decade in healthy individuals [43]. MacDonald et al. [44] used position emission tomography (PET) and found that increased reaction times in the aged population is closely related to the loss of dopamine receptors. Using imaging techniques like PET, researchers have reported a strong correlation between level of striatal dopamine transmission and motor decline in balance and gait parameters [45,46]. Moreover, the dopaminergic system has proven to be associated also with fine motor control, especially in older adults [47,48]. It has been suggested that failure of the dopaminergic system in aging might underlie the reduced velocity and control of fine movements [48]. Other researchers suggested that the alterations of the dopaminergic system can be due to the reduced inhibitory modulation which is regulated by the GABA neurons [40]. Inhibition of dopamine release and glutamate uptake occurring could potentially affect the ability to produce force and to preserve motor control in the elderly [11].

The GABAergic inhibitory system also plays an important role in isolating movements and retain brain neuroplasticity. A few investigators have suggested that neuroplasticity can be preserved almost entirely in the ageing brain [49,50,51,52]. Nevertheless, many researchers have reported that brain neuroplastic and neuromodulatory capabilities substantially decrease in the elderly population [53,54,55]. The GABAergic inhibitory system has been generally analysed in research using transcranial magnetic stimulation (TMS) [56,57], which has been proven to be a reliable method to assess neural inhibition and cortical excitability. Many TMS studies evidenced that the elderly population is characterised by a severely impaired sensorimotor integration of afferent input [54,55,56,58]. Fujiyama et al. [55] showed that a reduced capacity of GABA-mediated inhibition has a clear impact on short-interval intracortical inhibition during response preparation for a motor exercise. These findings were more recently supported by those of other authors [59,60,61,62,63,64]. Nonetheless, the effect of ageing on the GABAergic system has raised many questions and many discrepancies have emerged in the literature as recently described in a comprehensive review [65].

As previously stated, several TMS studies reported that ageing is associated with a decline in intracortical inhibition. Conversely, three studies reported an increased inhibition in older adults [66,67,68]. Furthermore, other researchers found very little to no effect of ageing on intracortical inhibition [54,69,70,71]. These inconsistencies between studies may be related to methodological differences, such as different TMS protocols, as well as differences between resting-state and task-related TMS assessments. To improve the interpretation of the cortical inhibitory-excitatory circuits, the TMS technique has been recently combined with electroencephalography (EEG) as in the study of Opie et al. [68] or brain imaging techniques such as magnetic resonance spectrometry (MRS) [65] and functional magnetic resonance imaging (fMRI), although the latter is limited by the inability to distinguish between inhibition and excitation [72].

Although GABAergic system failures can represent a valid and plausible reason to the age-related loss in cortical plasticity and corticospinal excitability, other factors, such as a decline in long-term potentiation [59] or altered gene expression [73], must be considered and further analysed.

## 3. Spinal Age-Related Adaptation

Advanced age is accompanied by a spinal neurodegenerative process, which includes both a structural and functional reorganisation at the spinal level. The most critical morphological alteration occurring in the spinal region is represented by spinal atrophy. This is primarily related to the loss of spinal motor neurons due to the apoptosis of the neural cells. In addition, the loss of motor neurons appears to be associated with an increase in the number of astrocytes as well as an alteration of the dendritic networks [74]. Several human studies also reported a reduction in the density and diameter of myelinated and unmyelinated axons in the ventral horns of the spinal cord of aged individuals [75,76,77,78,79,80]. Jacobs and Love [81] demonstrated that old adults show a decline in myelinated and unmyelinated fibres of 38% compared to young adults. Similarly, a study performed on aged rodents revealed that ageing is characterised by a loss of around 40% in the myelinated and unmyelinated fibres compared to young individuals [82]. Consistently, these authors reported a reduced axonal density and myelin thickness along with a considerable infiltration of connective tissue and increase of infolded and outfolded myelin loops [81,82,83].

One of the reasons for the age-related neurodegeneration at the spinal level has been attributed to a reduction of the endocrine and paracrine production of insulin-like growth factor-1 (IGF-1). IGF-1 provides not only an effective prevention mechanism, but also a compensation mechanism for the loss of spinal motor neurons with advancing age. Investigators have shown that IGF-1 plays an important role in motor neuron apoptosis, motor axon myelination, stimulation of axonal sprouting and repair of axons [84]. Although the mechanism underpinning the reduction of IGF-1 is still poorly understood, it appears that the inflammatory response which characterised the majority of elderly individuals may affect the local production of IGF-1. Grounds [85] observed an elevated level of inflammatory cytokines TNF-a and TNF-b in older adults and reported a strong correlation between the inflammatory response and the impaired IGF-1-mediated effects on motor neuron regeneration and axonal repair. The importance of IGF-1 on facilitating axonal sprouting has been demonstrated in animal experiments. In a study on young mice, a higher concentration of IGF-1 was accompanied by a greater capacity to reinnervate denervated muscle fibres after motor neurons loss [86]. The authors claimed that reinnervation through axonal sprouting can compensate for the loss of almost 50% of the original motor neurons.

Disfunction of spinal motor neurons, as well as reduced axonal myelination and reduced internodal length, have been considered responsible, at least in part, for the age-related decrease in nerve conduction velocity. Reductions in peripheral efferent and afferent axon action potential conduction velocity have been often reported in the literature [82,87,88,89,90,91]. Rivner et al. [92] demonstrated that advancing age highly correlates with variations of nerve conduction velocity and both motor and sensory responses. The analysis of the Hoffman (H) reflex via electromyography (EMG) and nerve stimulation has been proven to be a valid method to assess the efficacy of spinal circuitry function [93]. H-reflex primarily indicates the efficacy of Ia sensory afferent fibres to activate spinal motor neurons. Furthermore, EMG and nerve stimulation allow the analysis of the motor (M) wave which measures the direct activation of peripheral motor axons and, therefore, the magnitude of the motor response.

Many investigators revealed a significant reduction in the amplitude of the H-reflex response in old compared to young individuals. [94,95,96,97,98,99] This suggests that ageing may determine a decline in spinal motor neurons excitability although other factors such as pre-synaptic inhibition must be taken in consideration [100]. Along with a reduced H-reflex amplitude, the authors demonstrated that ageing is associated with an increased H-reflex latency [89,98]. These findings may help interpret the decline in peripheral nerve conduction velocity occurring at an advanced age. Interestingly, the research of Scaglioni et al. [89] revealed that M-wave latency was not different between young and aged individuals, in contrast to what emerged for the H-reflex latency. The same authors suggested that ageing could, therefore, affect sensory afferent fibres to a greater extent compared to efferent motor axons [89].

Another mechanism that seems to contribute to the spinal circuitry failures in aged individuals is the impaired modulation of the pre- and post-synaptic spinal inhibition [101]. Some investigators indicated that healthy young adults increase their muscle force by down-regulating their pre-synaptic inhibition, which, in turn, leads to enhanced excitatory afferent input. The research of Earles and co-workers [96] demonstrated that during an isometric voluntary contraction of a leg muscle, older participants exhibited a reduced modulation of spinal pre-synaptic inhibition although they were able to modulate the force similarly to young participants. Similarly, the work of Baudry et al. [102] indicated that older individuals did not modulate the amount of pre-synaptic inhibition of Ia afferents during a wrist extension task but rather increased the coactivation of the antagonist muscle. This appears to be related to a deterioration of the spinal afferent input and suggests that the elderly tend to rely less on spinal mechanisms and more on supraspinal mechanism in order to increase force [103].

## 4. Neuromuscular Junction

The progressive neuromuscular decline during ageing, as previously stated, can be accompanied by failures of the de-innervation–re-innervation mechanism, which normally compensates for neuronal loss and the related impairment in muscle strength and control. It appears that age-related neuromuscular junction (NMJ) dysfunction can primarily explain the progressive decline of the re-innervation process. As the majority of the human structures, also NMJ occurs in morphological remodelling and functional impairment as individuals age. Structural changes generally occur in the pre-synaptic area (motor nerve terminal) and post-synaptic area (muscle fibre surface and membrane), where the number of post-junctional folds in the motor endplate is significantly reduced, resulting in slower conduction velocity and decreased magnitude of the muscle action potentials [104,105]. Alterations of the nerve terminal region primarily include a numerical reduction of mitochondria in the plaque of the motor neuron terminal bouton. Mitochondria play a fundamental role in regulating metabolism, signal transduction and cell apoptosis and produce oxygen free radicals [106]. In addition, axonal mitochondria also function as a buffer for the calcium ion loads essential for excitation–contraction coupling [107].

Investigators have reported several morphological adaptations of the axonal mitochondria. Garcia et al. [108] indicated that mitochondria in the NMJ of the ageing population may incur cristae disruption, swelling and multiple fusions [109]. Studies regarding the pre-synaptic plaque have shown high levels of oxidative damage and nitrosylation. This appears to be directly responsible for the deterioration of the pre-synaptic mitochondria [110]. Some authors suggested that in the motor nerve terminals and the post-synaptic endplate, which are highly metabolically active, mitochondrial dysfunction may induce an even greater impairment [111,112]. In addition, Ibebunjo et al. [113] have recently indicated the presence of downregulation of mitochondrial energy metabolism in rats with NMJ disruption. However, the impact of oxidative stress on age-related adaptations of the peripheral nervous system remains still unclear and requires further investigation [108,114].

An author reported that in aged mitochondria located in the NMJ, there is evidence of altered calcium buffering and reduced ATP production, which may negatively affect both neurotransmission and vesicular recycling [115]. This suggests that the impaired excitation–contraction coupling occurring in elderly individuals could be closely related to the dysfunction of axonal mitochondria. However, some investigators have argued that the neurotransmission dysfunction is mainly associated with the age-related reorganisation of neurotransmitters receptors such as nicotine acetylcholine receptors (nAChR), which are found in the post-synaptic membrane, dihydropyridine receptors (DHPRs), which are located in the sarcolemma, and ryanodine receptors (RyRs) of the sarcoplasmic reticulum [116]. In particular, excitation–contraction uncoupling appears to be related to the mismatch between DHPRs and RyRs found in aged individuals. The interaction between DHPRs and RYRs plays a crucial role in the regulation of the calcium ions during contraction stimuli. Severe dysfunctions of these two receptors lead, in turn, to a decreased calcium release after an action potential, thus an impaired contraction [117,118].

Delbono [119] indicated that IGF-1 has a role in preventing the age-related failure of neurotransmitters receptors. A recent animal study suggested that overexpression of IGF-1 in mice can significantly reverse the numerical decrease of DHP receptors, thus preventing the dysfunction of the post-synaptic NMJ [120]. As previously described at the spinal level, there is strong evidence that IGF-1 can improve nerve regeneration and prevent neuronal loss. Moreover, many authors have highlighted the importance of IGF-1 in maintaining the integrity of the NMJ [121] and promote the re-innervation of previously de-innervated motor units in aged individuals [122].

The age-related structural and functional adaptations occurring at the NMJ have been further associated with other neuromuscular alterations such as reduced number of synaptic vesicles [123], reduced amounts of released neurotransmitters [115], a decline in satellite cell proliferation [124] and fragmentation of Schwann cells [125,126]. Several recent studies on Schwann cells emphasised their critical importance in neurodegenerative prevention, particularly concerning the NMJ synapse. In light of their capacity to regulate axonal regeneration, assist neural re-myelination fibres and provide functional recovery, degeneration of these cells might strongly contribute to ineffective re-innervation and neuromuscular dysfunction in ageing [127,128,129].

Authors have suggested that many NMJ impairments can be related to the high level of circulating inflammatory markers, such as cytokines and interleukines, which are commonly found in aged individuals. Elderly people generally incur chronic low-grade inflammation, also referred to as “inflammaging” [130], which represent a considerable risk factor for an accelerated decline of the neuromuscular structures, including NMJ. Previous literature clearly indicated that individuals suffering from chronic inflammation show evidence of muscle wasting and weakness [131,132]. In the NMJ, two mechanisms have been mainly identified in association with ageing inflammation. In the work of Saheb-Al Zamani et al. [133], overexpression of the interleukine 6 (IL-6) was found closely correlated with degeneration of Schwann cells in elderly, underlining the negative effect of inflammation on axonal regeneration. Furthermore, as reported in the previous paragraph, high levels of cytokines (TNF-a and TNF-b) in the aged population appear to down-regulate the production of IGF-1 and impair its regeneration activity [84].

Although there is indisputable evidence that elderly people present signs of muscle denervation and NMJ dysfunction, there is still no clear agreement if this process anticipates muscle sarcopenia or is a result of the decline of muscle fibres. Unfortunately, direct studies of the NMJ in humans are extremely challenging due to the delicate accessibility of its structures. The animal studies reported in this review have always required invasive surgical interventions (such as muscle needle biopsies) which are not commonly performed for research purposes in humans.

In living humans, insight into the NMJ decline during ageing is mostly reported via EMG assessments of single motor unit action potentials [134]. Current works on ageing NMJ have revealed severe alterations in the EMG recordings of single motor units. These consisted of abnormally large intervals between action potentials of two fibres of the same motor unit (Jitter) and higher variability in the shape of a single motor unit during consecutive discharges (Jiggle) [135,136]. Developing new neurophysiological techniques could be key to fully understanding the mechanisms underpinning the age-related neurodegeneration of the human NMJ. Substantial signs of progress have been made in the understanding of the molecular basis behind NMJ dysfunction through the analysis of circulating biomarkers as addressed in this paragraph. This area of investigation has a strong translational potential although the role of biomarkers, and their correlation with ageing neurodegeneration is not fully understood.

## 5. Muscle Fibre

Motor unit remodelling characterises elderly individuals and results in a considerable loss of innervated muscle fibres and a decrease in active fibres size [136,137]. Rates of fibres denervation greatly overcome re-innervation, and the decline in fibre size is closely related to the increase of oxidative stress and cell apoptosis, with significant reductions in the number of satellite cell responsible for muscle fibres regeneration [138,139].

Old humans generally exhibit muscle atrophy, which consists of smaller fibres in the active motor units compared to young adults. [140,141,142]. Reduced fibre cross-sectional area appears to occur across all fibres, although studies have reported contrasting results with significant variability determined by muscle type and sex of participants [137,143]. Age-related atrophy has been often associated with decreased protein synthesis and fewer satellite cells particularly in type II fibres [144,145,146]. In addition, histochemical studies in both humans and animals showed that older individuals tend to exhibit multiple myosin heavy chain (MHC) isoforms in single fibres [147]. The expression of multiple MHC isoform in older adults could often impede the conventional categorisation of muscle fibres in type I and type II.

Some studies indicated that muscle-specific tension may be retained with ageing despite the decrease in muscle fibre size [142,148]. Nonetheless, several works on old and very old individuals reported lower specific tension across different muscles at advancing age, especially in participants of 80 years or more [149,150]. The authors suggested that lower specific tension could be related to a decreased level of intracellular calcium and calcium sensitivity which characterise older individuals [151,152]. Animal studies have shown reductions of specific tension associated with excitation coupling impairments as comprehensively reviewed by Delbono [153]. The discrepancies in the findings on specific tension of single fibres in aged muscles seem to be related to sampling bias due to neglect of lifestyle modifiers such as physical activity and nutrition. Physical activity appears to strongly influence specific tension [150], thus studies with participants matched for physical activity are required to better understand the extent of specific tension decline at advancing age.

In old individuals, contractile properties of the muscle fibres equally decline as age increases [141]. In particular, old adults show reduced contractile speed compared with young people with several studies reporting lower rates of force development [154] and decreased maximal shortening velocity in single muscle fibres [149,155]. This decline has normally been associated with an impairment of the cross-bridge kinetics [143]. Authors have suggested that any age-related shift from type II to type I fibres can induce a compelling reduction in peak power and contractile speed up to six times lower compared to young adults [148].

Similar to contractile speed, also the rate of muscle relaxation appears to be reduced with ageing [156,157,158]. Authors indicated that alterations of the cross-bridge mechanics as well as reduced calcium uptake and calcium-ATPase activity are primarily responsible for impaired muscle relaxation in older people [159].

## 6. Conclusions and Perspectives

In this review, we analysed the most updated literature regarding central and peripheral adaptation occurring during the ageing process. The increased aged population has required and requires a more comprehensive understanding of the mechanisms involved in muscular decline due to its strong correlation with disability and mortality. When possible, we have highlighted the studies in which physiological changes were associated with functional outcomes.

The emerging picture is that ageing determines a structural and functional reorganisation at the central and peripheral level which, in turn, causes impairments in voluntary activation capacity and reduction in motor performance. Although studies have reported large variability in voluntary activation within older adults, there is evidence that elderly individuals show impairments in voluntary activation which varies in magnitude depending on the task performed and the muscle groups involved [160,161,162].

Neural factors, such as cortical adaptations appear to play a fundamental role in the deterioration of muscle quality, although a strong theoretical rationale often has not been accompanied by equally valuable evidence. Whilst the majority of scientists widely agrees on the structural remodelling of the ageing brain and the resulting motor impairments, further research is required to fully understand whether age-related reorganisation of inhibitory and excitatory circuits derive from neural malfunction or serve as a compensatory mechanism to preserve motor control in older individuals. Unanswered questions and literature inconsistencies may be addressed by combining newly developed diagnostics techniques and optimised procedures such as TMS in combination with novel brain imaging techniques (fMRI, MRS, magnetoencephalography) and electrophysiological monitoring techniques as, for instance, high-resolution EEG [163].

Age-related impairments occurring at the spinal level and in the NMJ area have been extensively analysed in the past and more recent research, but yet, a lack of agreement has arisen between different investigators. Introducing novel neurophysiological techniques as well as improving the understanding of the relationship between neuromuscular mechanisms such as pre- and post-synaptic inhibition and muscular voluntary activation could help resolve the discrepancies emerged in the literature. As previously described, the analysis of circulating biomarkers has carried a notable insight on the molecular basis behind the neurodegenerative process involving the spinal circuitry function and the NMJ. Further research is required to fully understand this process and the related mechanisms, in consideration of the strong translational potential of this area of research.

In order to understand the neuromuscular adaptations occurring at advancing age, several researchers have suggested the need to analyse the changes in neural drive to the muscles [164]. Surface EMG can provide some information on the neural drive to the muscles although the conventional procedures have been long debated and led to controversial conclusions, mainly due to the unavailability of motor unit population data. Recently, a novel, high-density EMG technique has been introduced to improve the estimation of the neural drive to the muscles [165]. Through the use of an array of electrodes rather than the usual bipolar configuration, a more accurate decomposition of the EMG signal appears to be feasible and it has shown promising results with regards to neural drive and motor unit properties estimation. Only few studies that used high-density EMG have been performed on elderly adults [166,167]. They reported evidence of impaired motor unit discharge characteristics which indicates a reduced integrity of the motor unit firing modulation [165,166]. Developing this technique could be key to more extensively interpreting the neural and intrinsic changes occurring with ageing.
